# Psychometric properties of a Saudi Arabian version of the Positive Mental Health (PMH) scale

**DOI:** 10.1186/s41155-022-00232-0

**Published:** 2022-09-20

**Authors:** Abdulmohsen Almubaddel

**Affiliations:** Department of Psychology, King Saud University, Riyadh, Saudi Arabia

**Keywords:** Positive mental health, Positive psychology, Validity, Reliability, Well-being, Scale validation, Happiness, Arabic, Factor analyses, Beck depression

## Abstract

The Positive Mental Health (PMH) scale has been shown to be a reliable and valid tool for assessing positive mental health and well-being in different languages and cultures. However, the PMH scale has not yet been translated into Arabic and validated for the Saudi Arabian population. Therefore, the current study aimed to translate the English version of the PMH scale into Arabic for the Saudi Arabian context and validate the translated scale. A total of 1148 adult participants from Saudi public universities took part in the study. Based on exploratory and confirmatory factor analyses in different subsamples, the results of the current study revealed that the unifactorial model satisfactorily fits the data. Additionally, the Arabic version of the PMH scale demonstrated sufficient levels of reliability and had a high negative correlation with the Beck Depression Inventory-II, indicating convergent validity. Taken together, the findings of the current study suggest that the Arabic version of the PMH scale has appropriate levels of validity and reliability for the Saudi Arabian population.

## Introduction

In recent years, the focus on mental health care has shifted from solely treating mental disorders to enhancing the positive aspects of mental health. A new goal in mental health care is the promotion of well-being. Long-term research shows that a high level of psychological well-being protects against mental illness and psychopathology and is also related to biological symptoms of physical health, reducing risks for various diseases (Weiss et al., 2016). Mental health studies have mostly focused on negative characteristics, such as health problems and mental disorders. However, some positive elements, including life satisfaction, social support, self-esteem, resilience, and happiness, are also being studied in contemporary research (Çeçen & Vatandaşlar, 2021; Diener et al., 2010; Seligman & Csikszentmihalyi, 2014). These positive elements of mental health are emphasized in the World Health Organization’s (World Health Organization, 2020) definition of health as “a state of complete physical, mental and social well-being and is not just the absence of disease or infirmity” (p. 1). This definition has broadened the focus on health from the absence of negative symptoms to the existence of well-being. Thus, defining mental health in terms of the nonexistence of a mental disorder is insufficient. In addition, research shows that the absence of positive mental health does not imply the presence of mental disorders.

Some studies indicate that positive mental health reduces the risk of mental disorders and boosts physical health and academic achievement (Keyes, 2005; Keyes & Simoes, 2012; Suldo & Shaffer, 2008). Promoting positive mental health in the work environment can help reduce absenteeism from work and increase productivity, especially among people with mental disorders (Zechmeister et al., 2008). Furthermore, boosting positive mental health also has economic benefits, such as helping lower the cost of psychotherapy, since fewer sessions are needed (Knapp et al., 2011; McDaid et al., 2019). Such initiatives require valid, reliable scales of positive mental health assessment that help in tracking a population’s mental health status. These scales can be used to collect data from different individuals and subgroups of a population and thus become critical in reviews of current mental health policies and services.

There are many scales that measure the level of positive mental health, including the Warwick–Edinburgh Mental Well-Being Scale (Tennant et al., 2007), Flourishing Scale (Diener et al., 2010), Flourishing Scale (Mesurado et al., 2021), Mental Health Continuum Scale (Keyes, 2002), Mental Health Continuum-Short Form (MHC-SF; Keyes, 2002; Lamers et al., 2011), General Health Questionnaire (GHQ; Hu et al., 2007), Life Orientation Test (Scheier & Carver, 1985), and Depression–Happiness Scale (McGreal & Joseph, 1993). Although all of these tools are generally characterized by adequate psychometric properties, some of them are multidimensional, while others assess specific concepts.

Addressing the need for a short scale to measure positive mental health using a comprehensive and non-multidimensional concept, Lukat et al. (2016) developed the Positive Mental Health (PMH) scale, which is a one-dimensional 9-item self-report scale with a more holistic view; a scale’s unidimensionality (Slocum-Gori & Zumbo, 2011) ensures that it measures a single concept such as positive mental health. The authors derived the nine items from other German scales: Lutz’s item pool, the Freiburg Personality Inventory (FPI-R), the mental health scale (SPG), and the Trier Personality Inventory (TPI). A feature of the PMH scale is that its items focus on an individual’s consistently stable judgments rather than on behavior in several varied situations. The items of the PMH scale are scored on a 4-point Likert scale ranging from 0 (not true) to 3 (true). All items are expressed positively. A high score on the scale indicates a high level of positive mental health.

Lukat et al. (2016) examined the psychometric properties of the PMH scale across a variety of samples (students [*N* = 5406], the general population [*N* = 1394], and clinicians [*N* = 1547]) and confirmed its unidimensionality. The authors also used depression scales that were theoretically expected to correlate negatively with the PMH scale to determine the latter’s convergent validity. The correlation coefficient values for these scales were as follows: −0.74 for the Depression Anxiety Stress Scale-21 (DASS-21), −0.57 to −0.71 for the Center for Epidemiological Studies Depression (CES-D) scale, and −0.53 to −0.68 for the Beck Depression Inventory (BDI). Concerning Cronbach’s alpha, Lukat et al. (2016) reported estimates ranging between 0.82 and 0.93, revealing high internal consistency. These results indicate that the PMH scale had satisfactory validity and reliability levels for the populations studied.

The PMH scale has since been adapted to and used in various cultures and languages; for instance, it has been employed in Germany, America, China, Russia, Pakistan, and Turkey (Bibi et al., 2020; Bieda et al., 2017; Cai et al., 2017; Çeçen & Vatandaşlar, 2021; Lin et al., 2019; Lukat et al., 2016; Margraf et al., 2016; Siegmann et al., 2018; Teismann, Brailovskaia, et al., 2018). Furthermore, several researchers have found support for the unidimensional construct validity of the scale, using confirmatory factor analysis (CFA) to verify the resultant model from exploratory factor analysis (EFA) and its reported measurement invariance across German, Russian, and Chinese cultures (Bieda et al., 2017), between Pakistanis and Germans (Bibi et al., 2020), and among Turkish students (Çeçen & Vatandaşlar, 2021). These authors have confirmed the PMH scale’s convergent validity by examining its relationship with other scales used to measure depression, which is supposed to be negatively associated with positive mental health. For instance, the coefficients of correlation of the PMH scale with depression were found to be −0.54 and −0.72 (Teismann, Forkmann, et al., 2018), −0.71 (Bibi et al., 2020), and −0.41 (Çeçen & Vatandaşlar, 2021). Regarding reliability, the studies by Brailovskaia et al. (2018a, b), Teismann, Forkmann, et al. (2018), Yilmaz Akbaba and Eldeleklioğlu (2019), Margraf et al. (2022), and Çeçen and Vatandaşlar (2021) indicate high Cronbach’s alpha values of 0.94, 0.92, 0.92, 0.85, 0.91, and 0.89, respectively. These studies and adaptations facilitate cross-cultural comparisons and allow the PMH scale to be used as a global tool to measure positive mental health, especially if the latent scale structure is found to be constant across the targeted cultures.

While studies on positive mental health conducted in universities have focused on students, the mental health of faculty members and employees has not been given enough attention, despite faculty members and employees being part of an interactive environment. Therefore, the current study focused on three groups in a university environment—students, faculty members, and employees—and assessed their levels of mental health, considering its influence on their academic and job performance. Doing so also provided us with unique opportunities to understand how the mental health of faculty and staff could affect the mental health of students.

In the current study, the English version of the PMH scale was translated into Arabic and validated for the Saudi Arabian context. To the best of our knowledge, the scale had previously not been translated into Arabic or validated among the Saudi Arabian population.

## Methods

### Design and setting

A descriptive study was conducted and reported in accordance with the STROBE statement and a checklist of items that should be included in reports of cross-sectional studies (von Elm et al., 2007). The study tools, which included demographic information forms, the PMH scale, and the Beck Depression Inventory-II (BDI-II), were prepared in an electronic format using Google Forms. Participants were recruited through snowball sampling via email invitations. The digital link to the questionnaire was sent to individuals whose email addresses were available on university websites.

### Participants

Participants were all Arabic speakers and affiliated with universities located in different regions of Saudi Arabia. Regarding the selection criteria, all participants were required to have been students, faculty members, or employees at a university for at least 1 year prior to the study and to have a minimum age of 18 years. Participation in the study was voluntary and anonymous, and all participants provided informed consent prior to participation. The current study received ethical approval from the research ethics committee of the King Saud University and was conducted in accordance with the Helsinki Declaration. All information provided by the participants was collected in an unidentifiable form. Data were collected between 1 December 2019 and 1 February 2020.

### Sample characteristics

The initial sample comprised 1261 participants, but based on the selection criteria, 113 of them were excluded; therefore, the final sample included 1148 participants, with ages ranging between 18 and 70 (mean age 31.29 years; *SD* ± 11.84). Men accounted for 44.8% of the sample, while 55.2% of the sample was female. Furthermore, 294 (25.6%) participants were from universities located in the western region of Saudi Arabia, 461 (40.1%) were from the central region, 87 (7.6%) were from the east, 180 (15.7%) were from the north, and 126 (11.0%) were from the south. Among the participants, 598 were students (52.1%), 478 were faculty members (41.6%), and 72 were employees (6.3%). Participation in the study was voluntary. The sample characteristics are described in Table 1.

**Table 1:** Sample characteristics (full sample, *n* = 1148)

	*n*	(%)
Gender	Frequencies	
Male	514	(44.8)
Female	634	(55.2)
Total	1148	(100)
Region		
Western region	294	(25.6)
Central region	461	(40.1)
Eastern region	87	(7.6)
Northern region	180	(15.7)
Southern region	126	(11.0)
Total	1148	(100)
Occupation		
Student	598	(52.1)
Faculty member	478	(41.6)
Employee	72	(6.3)
Total	1148	(100)

### Measures

Participants completed a survey including two different scales and questions regarding demographics, such as their gender, age, occupation, and university details. Positive mental health was measured using the PMH scale (open access; Lukat et al., 2016), and depression was measured using an Arabic version of the BDI-II (Ghareeb, 2000).

#### The Arabic version of the Positive Mental Health scale

The PMH scale was used to measure psychological and subjective aspects of well-being. This instrument includes nine items rated on a 4-point Likert scale ranging from 0 (strongly disagree) to 3 (strongly agree). The Arabic version of the PMH scale was translated from the English version (obtained from https://www.kli.psy.ruhr-uni-bochum.de/dips-interv/klipsy/download/pmh/PMH_Scale_english.pdf) by the present researcher using the translation–back-translation procedure. Then, the preliminary Arabic translation was carefully evaluated by two Arab psychologists for comparability in terms of meaning with the original English version. Necessary revisions were subsequently carried out. Next, the translated Arabic items were shared with an Arab specialist competent in both languages, who was then asked to translate them back into English. Finally, the original English items of the PMH scale were compared with their back-translated counterparts, and no significant differences in meaning were found between the two versions.

#### The Arabic version of the Beck Depression Inventory-II (Ghareeb, 2000)

The Arabic version of the BDI-II is a widely used self-report instrument with satisfactory psychometric properties. It was developed by Ghareeb (2000), who tested it with Saudi participants, as well as participants from 17 other Arab groups. This measurement tool consists of 21 items, and participants respond to the items using a 4-point scale (0–3). The total score varies between 0 and 63, and a high total score indicates severe depressive symptoms. A meta-analytical evaluation of the scale over 25 years showed that its internal consistency was between 0.73 and 0.92 (Beck et al., 1988). The BDI-II has acceptable validity and reliability among the Arabic-speaking population. Alhomoud et al. (2018) assessed the reliability of the scale’s estimated internal consistency among the Saudi population and derived a Cronbach’s alpha value of 0.86. For the current study, the internal consistency reliability of the BDI-II was measured again, and Cronbach’s alpha was determined to be 0.89.

### Data analysis

Data analyses were performed using SPSS 25 and Amos 23. For the sample characteristics, mean values and standard deviations (SDs) for continuous variables and frequencies and percentages for nominal variables were calculated. The skewness and kurtosis of the PMH scale items were checked. For a normal distribution, the skewness and kurtosis have a value of 0, while any value below 2 suggests that the data are normally distributed (Groeneveld & Meeden, 1984). For each item-scale assignment of the PMH scale, item-total correlations were computed after correcting for item overlap. Item-total correlations ≥ 0.30 were defined as acceptable (Döring & Bortz, 2016).

Furthermore, the internal consistency of the PMH scale was evaluated by calculating Cronbach’s alpha, McDonald’s omega, and composite reliability; values ≥ 0.70 were accepted as indicating sufficient reliability (George & Mallery, 2003).

Scale validity was assessed using EFA, CFA, and convergent validity. The full sample was randomly divided into two subsamples; one was considered for the EFA and the other for the CFA. The EFA was performed using principal axis factoring (PAF) to determine underlying factors in the prepared 9-item scale. PAF was chosen as the extraction method for the EFA because by using it, one is better able to recover weak factors and determine the least number of factors that can account for the common variance of a set of variables (Mabel & Olayemi, 2020).

Structural equation modeling (SEM) was conducted in the CFA group using IBM SPSS AMOS 23.0. The goodness of fit was reviewed using the comparative fit index (CFI), goodness fit index (GFI), incremental fit index (IFI), normed fit index (NFI), and relative fit index (RFI); all these indices had values of 0.90 or above, indicating a good fit. Another fit index is the root-mean-square error of approximation (RMSEA); an RMSEA value between 0.05 and 0.08 indicates an acceptable fit, while a value less than 0.05 indicates a good fit (Byrne & Campbell, 1999). Furthermore, a CMIN/DF value < 5 indicates an acceptable fit (Marsh & Hocevar, 1985). The magnitude of the standardized coefficients should be 0.40 (Howard, 2016).

To assess the convergent validity of the PMH scale, its relationship with the Arabic version of the BDI-II and the significance of this relationship were examined using Pearson’s correlation (r).

## Results

### Reliability and item analysis

The full-sample results (*n* = 1148) based on the absolute values of skewness and kurtosis for a total PMH scale score indicated that the sample data were normally distributed (skewness = −0.35 and kurtosis = 0.58). Additionally, all items correlated with the total scale to a good degree; the correlation coefficients ranged between 0.42 and 0.67, as shown in Table 2.

**Table 2:** Item statistics for the Positive Mental Health (PMH) scale

PMH scale items		Mean	Std. deviation	Skewness	Kurtosis	Item-total correlation
PMH scale (total score)		17.75	4.64	−0.35	0.58	
1	I am often carefree and in good spirits	1.85	0.76	−0.60	0.31	0.60
2	I enjoy my life	1.92	0.73	−0.51	0.29	0.65
3	All in all, I am satisfied with my life	2.01	0.76	−0.61	0.28	0.67
4	In general, I am confident	2.06	0.72	−0.55	0.38	0.63
5	I manage well to fulfill my needs	2.16	0.61	−0.35	0.67	0.47
6	I am in good physical and emotional condition	1.76	0.81	−0.32	−0.31	0.62
7	I feel that I am actually well equipped to deal with life and its difficulties	2.01	0.76	−0.66	0.44	0.60
8	Much of what I do brings me joy	1.91	0.73	−0.29	−0.17	0.61
9	I am a calm, balanced human being	2.07	0.73	−0.61	0.44	0.42

Regarding the analyses of instrument reliability, the Cronbach’s alpha, McDonald’s omega, and composite reliability coefficients for the PMH scale were found to be 0.86, 0.85, and 0.87, respectively, with the full sample (*n* = 1148). Since a coefficient ≥ 0.70 is considered acceptable (George & Mallery, 2003), it was determined that the PMH scale scores were consistent.

### Exploratory factor analysis (subsample, n = 579)

The EFA was performed using principal axis factoring PAF to determine underlying factors in the prepared 9-item scale. The Kaiser–Meyer–Olkin (KMO) measure of sampling adequacy presented a value of 0.895, while the value for Bartlett’s test of sphericity analysis was 1895.35, with sig. = 0.000. The EFA revealed one factor with an eigenvalue > 1 (EFA subsample = 4.33, male group = 4.45, and female group = 4.25), thus explaining (EFA subsample = 48.17%, male group = 49.54%, and female group = 47.21%) the total variance (Table 3).

**Table 3:** Bartlett’s test values, Kaiser–Meyer–Olkin (KMO) measures, item factor loadings, eigenvalues, and total explained variance

PMH scale items		Item factor loadings		
		EFA subsample *n* = 579	Male *n* = 248	Female *n* = 331
Bartlett’s test values		1895.35	867.31	1046.78
KMO measures		0.89	0.88	0.89
1	I am often carefree and in good spirits	0.70	0.68	0.71
2	I enjoy my life	0.75	0.74	0.75
3	All in all, I am satisfied with my life	0.77	0.74	0.79
4	In general, I am confident	0.73	0.77	0.70
5	I manage well to fulfill my needs	0.58	0.65	0.53
6	I am in good physical and emotional condition	0.71	0.70	0.72
	I feel that I am actually well equipped to deal with life and its difficulties	0.70	0.70	0.70
8	Much of what I do brings me joy	0.71	0.71	0.72
9	I am a calm, balanced human being	0.52	0.59	0.47
Eigenvalue		4.33	4.45	4.25
Total variance explained (%)		48.17	49.54	47.21

### Confirmatory factor analysis (subsample, n = 569)

**Fig. 1:**
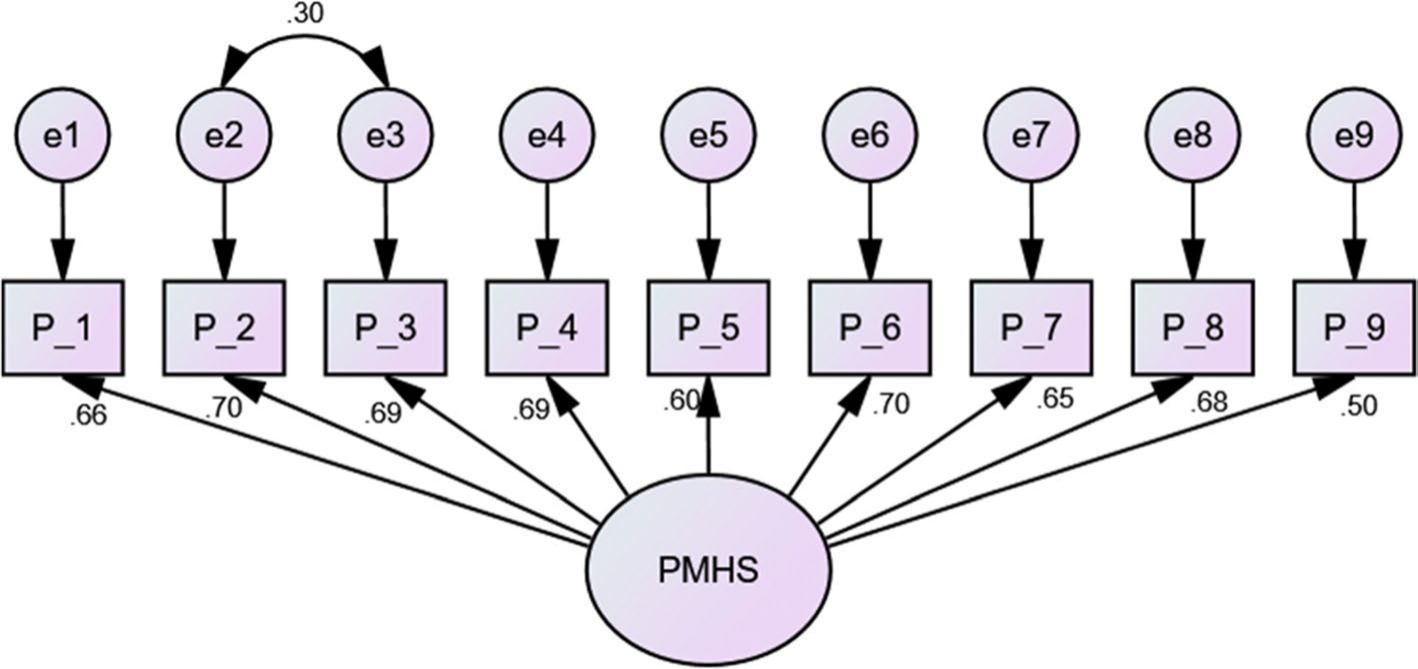
Confirmatory factor analysis model for the second subsample data, including standard loadings and standardized errors. *n* = 569. Final confirmatory factor analysis (CFA) model of the Positive Mental Health (PMH) scale

The one-factor solution determined via EFA was validated with the CFA subsample. The final SEM is shown in Fig. 1. The CFA confirmed the one-factor structure derived through EFA because all regression weights exhibited positive, highly significant (above 0.40), and highly satisfactory fit indices (Table 4).

**Table 4:** Fit indices of the confirmatory factor analysis (CFA) model with and without correlated errors

CFA subsample		CMIN/DF	NFI	CFI	TLI	IFI	RMR	RMSEA
All (*n* = 569)	Without correlated errors	5.56	0.92	0.93	0.91	0.92	0.2	0.09
	With correlated errors	4.30	0.94	0.95	0.93	0.95	0.02	0.07
Male (*n* = 266)	Without correlated errors	2.37	0.93	0.96	0.95	0.96	0.01	0.07
Female (*n* = 303)	Without correlated errors	4.99	0.85	0.87	0.83	0.88	0.03	0.11
	With correlated errors	2.76	0.92	0.95	0.92	0.95	0.02	0.07

**Fig. 2:**
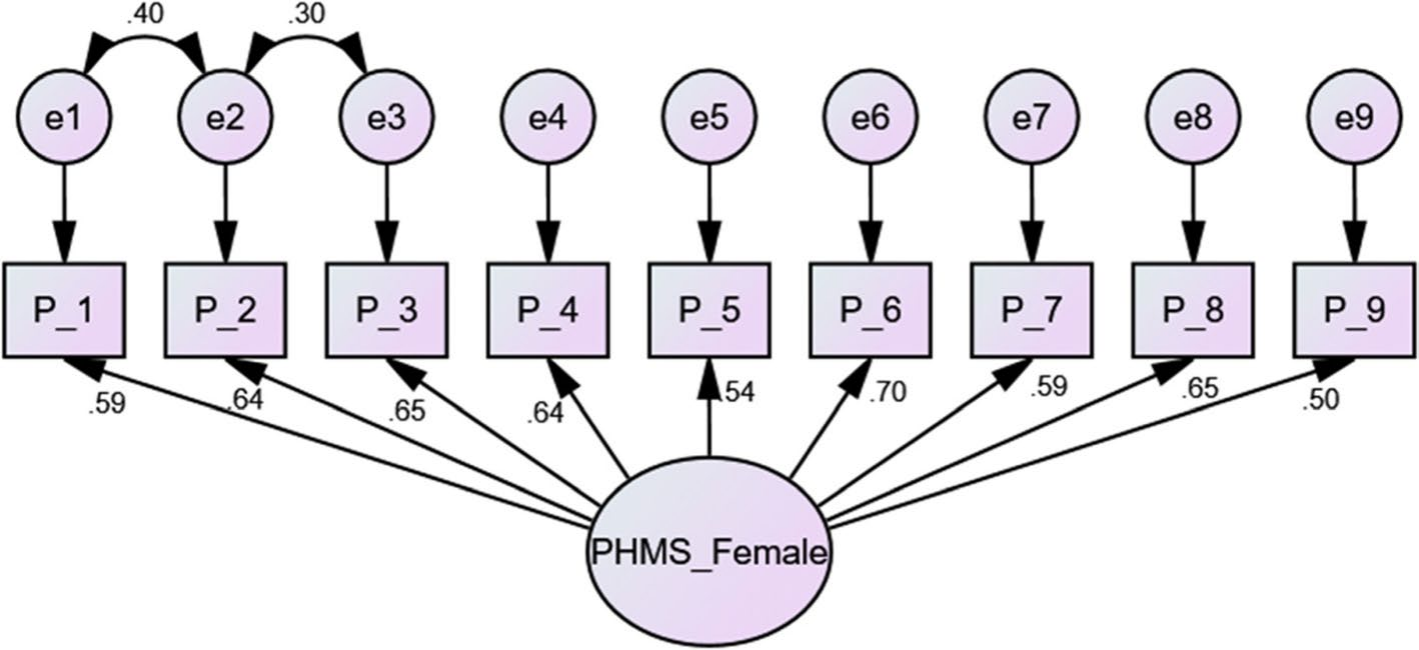
Confirmatory factor analysis model for female data from the second subsample data including standard loadings and standardized errors. *n* = 303. Final confirmatory factor analysis (CFA) model of the Positive Mental Health (PMH) scale (female)

**Fig. 3:**
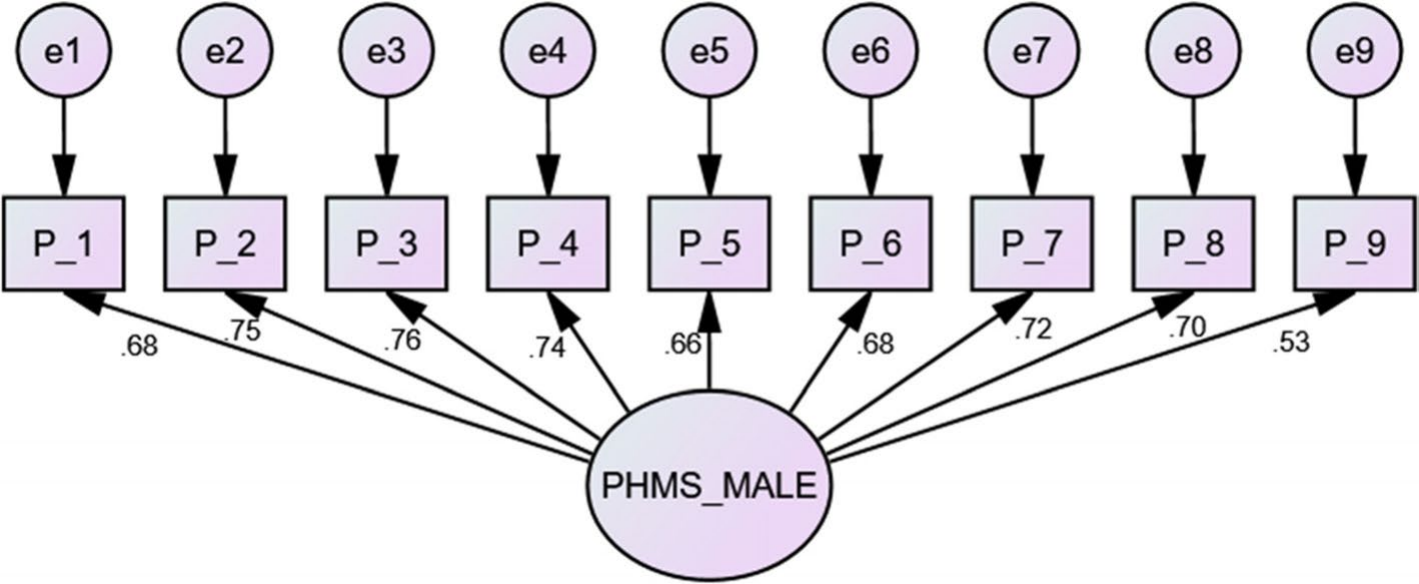
Confirmatory factor analysis model for male data from the second subsample data including standard loadings and standardized errors. *n* = 266. Final confirmatory factor analysis (CFA) model of the Positive Mental Health (PMH) scale (male)

Based on the modification indices, some error terms were correlated by adding covariance paths among the error terms for items 2 and 3 with the CFA subsample (Fig. 1) and among the error terms for items 1, 2, and 3 with the female group, which improved the fitting model (Fig. 2). In addition, no cross-loaded modification index or path between the error terms and items was determined in the male group (Fig. 3).

### Measurement invariance across genders (subsample, n = 569)

Based on the results of the CFA, the one-factor structure was used as the baseline model in the measurement invariance testing. The fit indices of the configural invariance model (*CMIN* = 158.29, *df* = 52, *CFI* = 0.94, *TLI* = 0.92, and *RMSEA* = 0.06) were all acceptable. In addition, both *Δ*CFI values between nested models were 0.001, which indicated that the one-factor structure reached strict invariance between men and women. The specific information is summarized in Table 5.

**Table 5:** Measurement invariance of the Positive Mental Health (PMH) scale across genders

Model	CMIN	DF	CMIN/DF	TLI	CFI	RMSEA (90% *CI*)	ΔCFI
Configural invariance	158.29	52	3.04	0.92	0.94	0.06 (0.049, 0.071)	
Metric invariance	164.181	60	2.73	0.93	0.94	0.05 (0.045, 0.066)	0.001
Scalar invariance	165.836	61	2.71	0.93	0.95	0.05 (0.045, 0.065)	0.001

### Convergent validity (full sample, *n* = 1148)

Convergent validity was confirmed with a moderate correlation coefficient (−0.65) between the Saudi Arabian version of the Positive Mental Health scale (PMH scale) and the Arabic version of the Beck Depression Inventory-II (BDI-II).

## Discussion

The current study aimed to translate and adapt the PMH scale to the Arabic language spoken in Saudi Arabia and investigate its psychometric properties in Saudi Arabian adults. The construct validity of the scale was assessed using EFA, CFA, and convergent validity, while the reliability and internal consistency of the scale were assessed using Cronbach’s alpha, McDonald’s omega, and composite reliability.

The findings show that the Arabic version of the scale had satisfactory psychometric properties with good reliability and validity. These findings are consistent with those corresponding to the original version of the scale (Lukat et al., 2016) and with the outcomes of other studies (Brailovskaia et al., 2018a, b; Çeçen & Vatandaşlar, 2021; Margraf et al., 2022; Teismann, Forkmann, et al., 2018; Yilmaz Akbaba & Eldeleklioğlu, 2019). They thus support the robustness and reliability of the instrument for assessing positive mental health in Saudi Arabia.

The EFA and CFA results support the unidimensional construct validity, largely resembling their counterparts in the majority of studies in which a one-factor structure was replicated (Bibi et al., 2020; Bieda et al., 2017; Çeçen & Vatandaşlar, 2021; Lukat et al., 2016; Yilmaz Akbaba & Eldeleklioğlu, 2019). Based on this finding, it is concluded that the PMH scale has good construct validity. In addition, this finding may indicate that positive mental health can be measured as a single, unidimensional concept, as shown by the male and female participants of this study.

Although the results of prior studies did not show the need to correlate error terms in the CFA, in the current study, error terms in the female sample had to be correlated for the model to be acceptable, while there was no need to correlate errors in the male sample. No convincing reason was detected to make the errors correlated between items 1 (I am often carefree and in good spirits) and 2 (I enjoy my life) and between items 2 and 3 (All in all, I am satisfied with my life) in the female sample for the model to be accepted. Perhaps, this can be explained by the fact that items 1, 2, and 3 refer to concepts of carefreeness, enjoyment, and satisfaction, respectively. These themes may be similar in the perceptions of females but not in those of males. In other words, men and women need not share the same perceptions of carefreeness, enjoyment, and satisfaction, which can be reasonably explained by a correlated error or an additional factor for the female sample. This indicates the possibility of a difference in the latent structure of the PMH scale between men and women when repeating the study in Saudi society.

To examine whether the one-factor structure reached measurement invariance across genders, the configural invariance model, metric invariance model, scalar invariance model, and strict invariance model were tested. Subsequently, it was found that the one-factor structure achieved strict gender invariance after the error terms were correlated.

Furthermore, the Arabic version of the PMH scale was found to have a high negative correlation with the BDI-II—it correlated significantly with depression in the expected direction, providing evidence of convergent validity. This result is in line with the findings of a validation study conducted by Lukat et al. (2016), who found strong negative relationships between positive mental health and depression. Multiple studies have also reported that the PMH scale scores are negatively related to the BDI-II scores of German, Pakistani, and Turkish students (Bibi et al., 2020; Çeçen & Vatandaşlar, 2021; Teismann, Forkmann, et al., 2018).

The participants of this study were recruited through snowball sampling, which may have given rise to sample selection bias. Additionally, the number of employees in the sample was small (70 employees) relative to the numbers of students and faculty members. However, the large sample size may have compensated for these two limitations. The findings should be viewed in light of the study focus being limited to students, faculty members, and employees. Therefore, the PMH scale may not be transferable to other staff, such as health care and security staff. Thus, the study findings are not generalizable to the overall Saudi population. Furthermore, another limitation is that only depression was used to assess convergent validity, which is only one aspect of negative mental health. Negative mental health is reflected in several constructs, such as anxiety and stress, so future studies should investigate additional tools to deepen our understanding of the scale.

Considering the relevance and worldwide application of the PMH scale, in future studies, items of the Arabic version of the scale that sound excessively similar or contain redundancy in meaning (e.g., the coupled items in the female sample data—items 1 and 2 and items 2 and 3) should be rephrased. Moreover, further research is needed to replicate our findings or apply the PMH scale in other contexts, preferably with different populations, such as participants from secondary schools, companies, and factories.

## Conclusion

The Arabic version of the PMH scale showed generally satisfactory psychometric properties when applied among Saudi Arabian students, faculty members, and university employees. Based on our findings, it is proposed that this version can be used for various purposes related to promoting positive mental health (e.g., in education, psychology, and psychological counseling) and for research comparing positive mental health and cultural factors. Nevertheless, generating more psychometric data on this scale by employing it in further studies with Saudi adults would be useful. The scale can be used as a quick screening instrument to assess positive mental health levels in educational and professional institutions, thereby helping promote positive mental health in the work environment.

## Data Availability

The data that support the findings of this study are available from the author upon reasonable request and are provided as electronic supplementary material.

## Abbreviations

PMH scale: Positive Mental Health scale
WHO: World Health Organization
BDI-II: Beck Depression Inventory-II
EFA: Exploratory factor analysis
PAF: Principal axis factoring
KMO: Kaiser–Meyer–Olkin
CFA: Confirmatory factor analysis
SEM: Structural equation modeling

## References

[CR1] Alhomoud MA, Gosadi IM, Wahbi HA (2018). Depression among sickle cell anemia patients in the Eastern Province of Saudi Arabia. Saudi Journal of Medicine & Medical Sciences.

[CR2] Beck AT, Steer RA, Carbin MG (1988). Psychometric properties of the Beck Depression Inventory: Twenty-five years of evaluation. Clinical Psychology Review.

[CR3] Bibi A, Lin M, Margraf J (2020). Salutogenic constructs across Pakistan and Germany: A cross sectional study. International Journal of Clinical and Health Psychology.

[CR4] Bieda A, Hirschfeld G, Schönfeld P, Brailovskaia J, Zhang XC, Margraf J (2017). Universal happiness? Cross-cultural measurement invariance of scales assessing positive mental health. Psychological Assessment.

[CR5] Brailovskaia J, Teismann T, Margraf J (2018). Cyberbullying, positive mental health and suicide ideation/behavior. Psychiatry Research.

[CR6] Brailovskaia J, Teismann T, Margraf J (2018). Physical activity mediates the association between daily stress and Facebook addiction disorder (FAD)—A longitudinal approach among German students. Computers in Human Behavior.

[CR7] Byrne BM, Campbell TL (1999). Cross-cultural comparisons and the presumption of equivalent measurement and theoretical structure: A look beneath the surface. Journal of Cross-Cultural Psychology.

[CR8] Cai D, Zhu M, Lin M, Zhang XC, Margraf J (2017). The bidirectional relationship between positive mental health and social rhythm in college students: A three-year longitudinal study. Frontiers in Psychology.

[CR9] Çeçen AR, Vatandaşlar SE (2021). Psychometric properties of the Positive Mental Health scale (PMH-scale) among Turkish university students. European Journal of Health Psychology.

[CR10] Diener E, Wirtz D, Tov W, Kim-Prieto C, Choi D, Oishi S, Biswas-Diener R (2010). New well-being measures: Short scales to assess flourishing and positive and negative feelings. Social Indicators Research.

[CR11] Döring N, Bortz J (2016). Forschungsmethoden und Evaluation in den Sozial- und Humanwissenschaften [Research methods and evaluation in the social and human sciences].

[CR12] George D, Mallery P (2003). SPSS for Windows step by step: A simple guide and reference: 11.0 update.

[CR13] Ghareeb A (2000). Manual of the Arabic BDI-II. Alongo Press. Cairo Inventory: The author’s twenty-five years of evaluation. Clinical Psychology Review.

[CR14] Groeneveld RA, Meeden G (1984). Measuring skewness and kurtosis. Journal of the Royal Statistical Society: Series D (The Statistician).

[CR15] Howard MC (2016). A review of exploratory factor analysis decisions and overview of current practices: What we are doing and how can we improve?. International Journal of Human-Computer Interaction.

[CR16] Hu Y, Stewart-Brown S, Twigg L, Weich S (2007). Can the 12-item General Health Questionnaire be used to measure positive mental health?. Psychological Medicine.

[CR17] Keyes CLM (2002). The mental health continuum: From languishing to flourishing in life. Journal of Health and Social Behavior.

[CR18] Keyes CLM (2005). Chronic physical conditions and aging: Is mental health a potential protective factor?. Ageing International.

[CR19] Keyes CLM, Simoes EJ (2012). To flourish or not: Positive mental health and all-cause mortality. American Journal of Public Health.

[CR20] Knapp M, McDaid D, Parsonage M (2011). Mental health promotion and mental illness prevention: The economic case.

[CR21] Lamers SMA, Westerhof GJ, Bohlmeijer ET, ten Klooster PM, Keyes CLM (2011). Evaluating the psychometric properties of the Mental Health Continuum-Short Form (MHC-SF). Journal of Clinical Psychology.

[CR22] Lin M, Hirschfeld G, Margraf J (2019). Brief form of the Perceived Social Support Questionnaire (F-SozU K-6): Validation, norms, and cross-cultural measurement invariance in the USA, Germany, Russia, and China. Psychological Assessment.

[CR23] Lukat J, Margraf J, Lutz R, van der Veld WM, Becker ES (2016). Psychometric properties of the Positive Mental Health scale (PMH-scale). BMC. Psychology.

[CR24] Mabel OA, Olayemi OS (2020). A comparison of principal component analysis, maximum likelihood and the principal axis in factor analysis. American Journal of Mathematics and Statistics.

[CR25] Margraf J, Lavallee K, Zhang X, Schneider S (2016). Social rhythm and mental health: A cross-cultural comparison. PLoS ONE.

[CR26] Margraf J, Lavallee KL, Zhang XC, Woike JK, Schneider S (2022). Mental health and the wish to have a child: A longitudinal, cross-cultural comparison between Germany and China. Journal of Psychosomatic Obstetrics & Gynecology.

[CR27] Marsh HW, Hocevar D (1985). Application of confirmatory factor analysis to the study of self-concept: First- and higher order factor models and their invariance across groups. Psychological Bulletin.

[CR28] McDaid D, Park A-L, Wahlbeck K (2019). The economic case for the prevention of mental illness. Annual Review of Public Health.

[CR29] McGreal R, Joseph S (1993). The Depression-Happiness Scale. Psychological Reports.

[CR30] Mesurado B, Crespo RF, Rodríguez O, Debeljuh P, Carlier SI (2021). The development and initial validation of a multidimensional flourishing scale. Current Psychology.

[CR31] Scheier MF, Carver CS (1985). Optimism, coping, and health: Assessment and implications of generalized outcome expectancies. Health Psychology.

[CR32] Seligman MEP, Csikszentmihalyi M (2014). Positive psychology: An introduction. Flow and the foundations of positive psychology: The collected works of Mihaly Csikszentmihalyi.

[CR33] Siegmann P, Teismann T, Fritsch N, Forkmann T, Glaesmer H, Zhang XC, Brailovskaia J, Margraf J (2018). Resilience to suicide ideation: A cross-cultural test of the buffering hypothesis. Clinical Psychology & Psychotherapy.

[CR34] Slocum-Gori SL, Zumbo BD (2011). Assessing the unidimensionality of psychological scales: Using multiple criteria from factor analysis. Social Indicators Research.

[CR35] Suldo SM, Shaffer EJ (2008). Looking beyond psychopathology: The dual-factor model of mental health in youth. School Psychology Review.

[CR36] Teismann T, Brailovskaia J, Siegmann P, Nyhuis P, Wolter M, Willutzki U (2018). Dual factor model of mental health: Co-occurrence of positive mental health and suicide ideation in inpatients and outpatients. Psychiatry Research.

[CR37] Teismann T, Forkmann T, Brailovskaia J, Siegmann P, Glaesmer H, Margraf J (2018). Positive mental health moderates the association between depression and suicide ideation: A longitudinal study. International Journal of Clinical and Health Psychology.

[CR38] Tennant R, Hiller L, Fishwick R, Platt S, Joseph S, Weich S, Parkinson J, Secker J, Stewart-Brown S (2007). The Warwick-Edinburgh Mental Well-Being Scale (WEMWBS): Development and UK validation. Health and Quality of Life Outcomes.

[CR39] von Elm E, Altman DG, Egger M, Pocock SJ, Gøtzsche PC, Vandenbroucke JP (2007). The Strengthening the Reporting of Observational Studies in Epidemiology (STROBE) statement: Guidelines for reporting observational studies. Bulletin of the World Health Organization.

[CR40] Weiss LA, Westerhof GJ, Bohlmeijer ET (2016). Can we increase psychological well-being? The effects of interventions on psychological well-being: A meta-analysis of randomized controlled trials. PLoS ONE.

[CR41] World Health Organization (2020). Basic documents.

[CR42] Yilmaz Akbaba A, Eldeleklioğlu J (2019). Adaptation of Positive Mental Health scale into Turkish: A validity and reliability study. Journal of Family Counseling and Education.

[CR43] Zechmeister I, Kilian R, McDaid D, the MHEEN group (2008). Is it worth investing in mental health promotion and prevention of mental illness? A systematic review of the evidence from economic evaluations. BMC Public Health.

